# Role of Intracellular Stochasticity in Biofilm Growth. Insights from Population Balance Modeling

**DOI:** 10.1371/journal.pone.0079196

**Published:** 2013-11-13

**Authors:** Che-Chi Shu, Anushree Chatterjee, Wei-Shou Hu, Doraiswami Ramkrishna

**Affiliations:** 1 School of Chemical Engineering, Purdue University, West Lafayette, Indiana, United States of America; 2 Department of Chemical Engineering and Materials Science, University of Minnesota, Minneapolis, Minnesota, United States of America; University of California San Diego, United States of America

## Abstract

There is increasing recognition that stochasticity involved in gene regulatory processes may help cells enhance the signal or synchronize expression for a group of genes. Thus the validity of the traditional deterministic approach to modeling the foregoing processes cannot be without exception. In this study, we identify a frequently encountered situation, i.e., the biofilm, which has in the past been persistently investigated with intracellular deterministic models in the literature. We show in this paper circumstances in which use of the intracellular deterministic model appears distinctly inappropriate. In *Enterococcus faecalis,* the horizontal gene transfer of plasmid spreads drug resistance. The induction of conjugation in planktonic and biofilm circumstances is examined here with stochastic as well as deterministic models. The stochastic model is formulated with the Chemical Master Equation (CME) for planktonic cells and Reaction-Diffusion Master Equation (RDME) for biofilm. The results show that although the deterministic model works well for the perfectly-mixed planktonic circumstance, it fails to predict the averaged behavior in the biofilm, a behavior that has come to be known as *stochastic focusing.* A notable finding from this work is that the interception of antagonistic feedback loops to signaling, accentuates stochastic focusing. Moreover, interestingly, increasing particle number of a control variable could lead to an even larger deviation. Intracellular stochasticity plays an important role in biofilm and we surmise by implications from the model, that cell populations may use it to minimize the influence from environmental fluctuation.

## Introduction

More than sixty percent of bacterial infections treated in hospitals involve biofilm formation in the body [1]. Biofilm is the consequence of bacteria encasing themselves in a slimy layer of extracellular hydrated polymer matrix secreted by them [2]. Pathogenic biofilm is notorious for its high resistance to antibiotics [3]–[5] and causing chronic infection [6]. It is possible that conjugation, one of the horizontal gene transfer processes, contributes to antibiotic resistance of the biofilm [7]. In this work, the induction of conjugative plasmid pCF10 encoding tetracycline resistance is studied as an example to illustrate the importance of considering intracellular stochasticity on formulating a mathematical model for the biofilm.

Research on modeling biofilms has been increasing steadily in the past few decades resulting in the elucidation of some features of the biofilm. The layer model, which is usually composed of a structure in which cells are distributed uniformly, is broadly applied to analyze the biofilm in a reactor [8]–[10]. The structural models which capture the variable biofilm thickness, density, porosity and surface shape are usually constructed with cellular automata [11]–[13] or particle-based model [14], [15]. The transfer of drug resistance [16] or spread of pathogen [17] has also been described by empirically assigning some factors to cells which may not be directly based on intracellular gene regulation. However, current biofilm models focus much more on extracellular structure and mass transfer than intracellular gene regulation; only a few of them incorporate stochasticity in intracellular processes.

Stochasticity in gene expression arises from randomness associated with cellular processes. Attention to fluctuations in intracellular concentrations has arisen out of their implications to gene regulation and stochastic as well as phenotypic variability [18]–[23]. The noise of gene regulation is characterized by appearance of a distribution of intracellular concentrations among a population. It is generally understood that a bimodal distribution of protein concentration may be observed when bistability is encountered in deterministic behavior [24], [25] although in light of [26], it should be recognized that single cell bistability does not always lead to a bimodal distribution in the population. The deterministic model fails to predict the average behavior for a system with bimodal distribution as it is unable to describe the switch from one mode to another. There also are other limitations of the deterministic model; recent findings such as stochastic resonance [27], [28], stochastic focusing [29], frequency-modulated synchronization [30], [31], and so on [32]–[34] also fall beyond the scope of the deterministic model. From all of the foregoing considerations, indiscriminate use of the deterministic model is ill-advised.

In the current study, we develop a detailed understanding of the deterministic model for describing gene regulatory phenomena in the biofilm by comparing it with a comprehensive stochastic model. Towards this end, we analyze the induction of conjugative plasmid, pCF10, in *Enterococcus faecalis* under both planktonic and biofilm circumstances. The model shows that the deterministic approach works well for planktonic situations but deviates seriously for biofilms. It becomes important to realize that the biofilm circumstance alters the nature of intracellular stochasticity which cannot be captured by the simplicity of a deterministic model.

## Models

### Mechanism of Conjugative Gene Regulation

**Figure 1:**
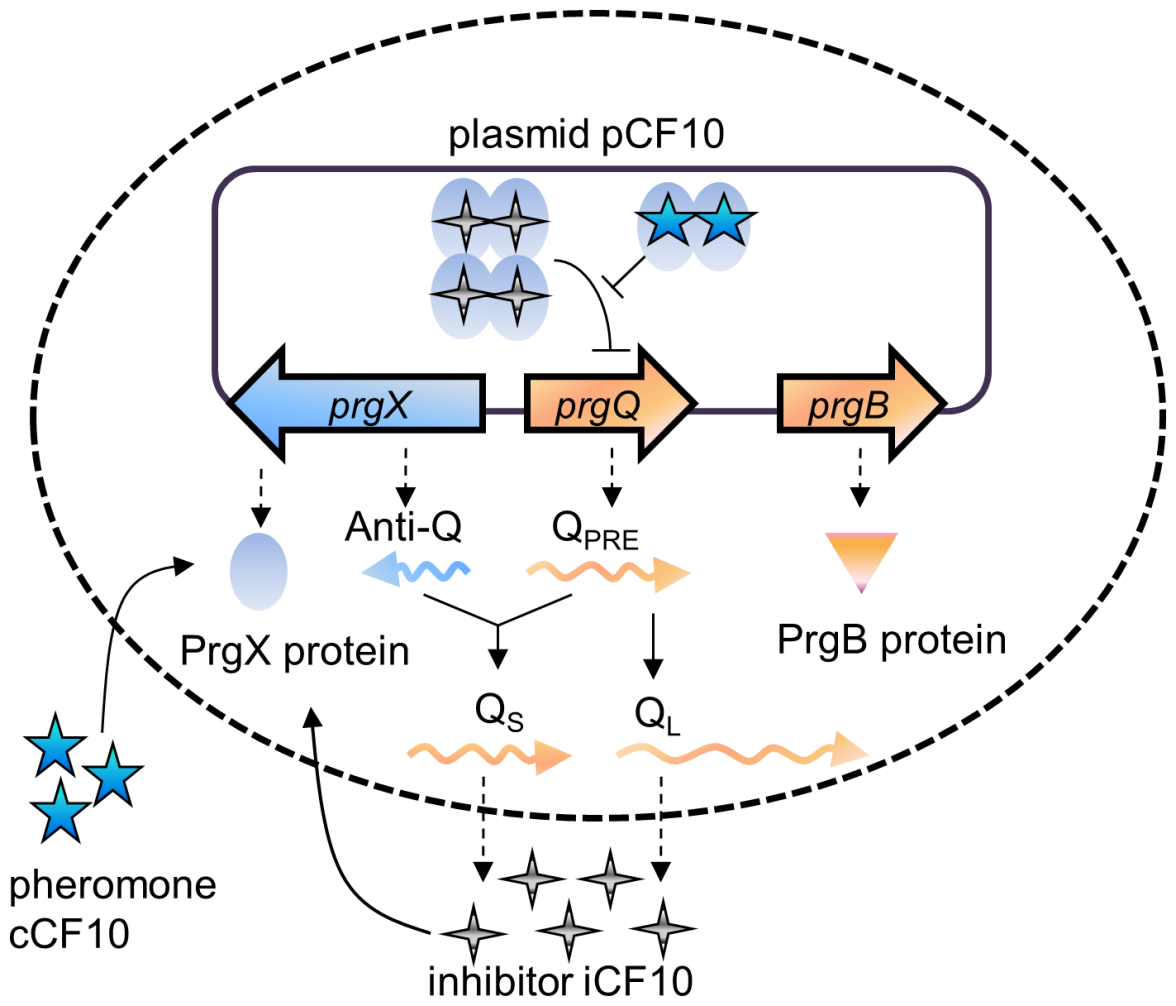
The gene regulation of pCF10 conjugation. The *prgQ-prgX* gene pair regulates conjugation. The inhibitor iCF10 released by pCF10 carrying donor cells, is encoded by Q_S_ and Q_L_ RNA, a product of the *prgQ* gene. The pheromone cCF10 is released by pCF10 deficient recipient cells in the extracellular environment or by added in. Without the presence of cCF10, iCF10-PrgX protein complexes bind to pCF10 DNA and repress the expression of *prgQ* gene. When cCF10 enters the donor cell, it alters the structure of PrgX protein and restores *prgQ* gene to active conformation. In the repressed conformation, nearly all Q_PRE_ reacts with Anti-Q to become Q_s_ RNA which has no effect on downstream conjugation-causing genes. In active conformation, overwhelming amount of Q_PRE_ results in the production of Q_L_ to stimulate the expression of *prgB.* The membrane protein PrgB helps pCF10 carrying donor cells transfer pCF10 to recipient cells.

The transfer of drug resistance in both planktonic and biofilm environments has been examined in this study. Plasmid pCF10, in *Enterococcus faecalis*, encoding tetracycline resistance is transferred from pCF10 carrying donor cells to pCF10 deficient recipient cells via inducible conjugation [35]. A signaling molecule, cCF10, secreted by recipient cells [36] or provided by external addition, triggers the intracellular gene regulation of donor cells to execute conjugation. In this study, no plasmid transfer is examined but only gene regulatory process has been investigated as it is the focus of many researchers [37]–[41]. The network of the gene regulatory process is shown in figure 1. Without cCF10, the plasmid DNA is bound with a tetramer of repressor PrgX, which hinders RNA polymerase binding to *prgQ* promoter and reduces the production of Q_PRE_ [42], [43], whose downstream products induce conjugative transfer of plasmid pCF10 between donor and recipient cells. In the opposite direction, Anti-Q is continually expressed [44]. Anti-Q binds to Q_PRE_ through sense-antisense interaction [44] and becomes Anti-Q:Q_s_ complex [45], [46], or simply Q_s_ in the model. The other longer mRNA, Q_L_, comes from Q_PRE_ with no Anti-Q bound. Both Q_s_ and Q_L_ mRNA are translated to produce iCF10, the other signaling molecule which behaves as an inhibitor to cCF10, but only Q_L_ stimulates the expression of downstream gene including *prgB* [47], an indicator for the onset of conjugation [39], [41]. Normally, without cCF10 the amount of Q_L_ is small. In the presence of cCF10, a PrgX tetramer breaks into two dimers that lift the repression on *prgQ* promoter [48]. When Q_PRE_ overwhelms Anti-Q, unbound Q_PRE_ starts to produce Q_L_ [49] which stimulates the expression of *prgB* encoding a surface aggregation protein and makes conjugation occur [47]. In a nutshell, plasmid DNA is normally bound with protein tetramer to stay at repressed configuration with less *prgQ* gene expression. Without enough Q_PRE_, the cell produces Q_s_ RNA which has no effect on downstream gene expression. In the opposite case, with cCF10, plasmid DNA gets rid of protein tetramer and changes to active conformation. Then, the cell produces Q_L_ RNA which stimulates the expression of *prgB* and enables conjugation.

### Modeling the Planktonic Environment

In the planktonic environment, the cells and extracellular variables are considered to be perfectly mixed. It will be of interest to consider both deterministic and stochastic models of gene regulation while accounting for the exchange of species between the cells and the environment.

#### Description of the deterministic model for planktonic environment

A detailed explanation of our notation is shown in Table 1. The set of intracellular concentration variables is represented by a vector 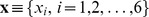, which contains as components, the concentrations of various intracellular species denoted by lower case letters in Table 1. The extracellular concentration variables are contained in a vector 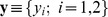. For the deterministic model the equations are formulated with the assumption of perfect mixing and using mass action kinetics. The dynamics associated with the intracellular variables through cellular processes and gene regulation is described by a rate vector 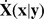 containing the deterministic reaction rates in terms of intracellular concentration vector 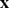 and extracellular concentration vector 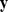
_,_ where the notation “ 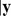 ” means “given 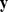
”. The rate of change of extracellular variables is denoted by the vector 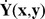. We also use particle numbers of each species and represent them by symbols with caret tops. Thus we use 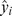 to denote the number of the 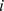 ^th^ extracellular species whose concentration is given by 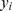,i.e., 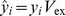. Further, the rate of change of extracellular variables in number is denoted by the vector 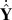.

**Table 1:** Nomenclature of pCF10 system.

SPECIES	SYMBOL	Intracellular concentration		Extracellular concentration	
		Component	Symbol	Component	Symbol
**mRNA**	Q_s_	*x*_1_	*q_s_*		
**mRNA**	Q_L_	*x*_2_	*q_L_*		
**Truncated** **RNA**	Anti-Q	*x*_3_	*q_a_*		
**Inhibitor**	*iCF*10	*x*_4_	*i*	*y*_1_	*I*
**Pheromone**	*cCF*10	*x*_5_	*c*	*y*_2_	*C*
**Protein**	PrgB	*x*_6_	*b*		

The set of differential equations describing intracellular or extracellular concentrations in vector form are shown below.
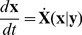
(1)

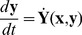
(2)



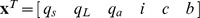
, 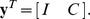


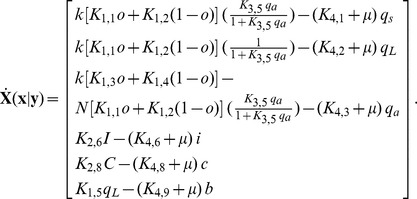
where 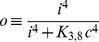, represents plasmid DNA in repressed form


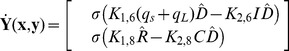
 where 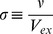 represents the ratio of the cell volume 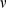 to the extracellular volume 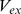. The entire nomenclature is shown in Table 2 with the values of the reaction constants in Table 3. 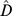 represents the total number of donor cells in the system and 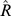, the total number of recipient cells. For simulation, the initial cell numbers and volume conversion factor are chosen as 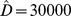, 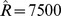, and 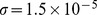. The initial conditions of intracellular states or extracellular variables are assumed to be the steady-state values of the pure donor culture and have been denoted as 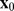 and 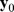.

**Table 2:** Nomenclature of pCF10 system.

Notation	Name
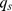	Intracellular concentration of Q_s_ mRNA
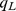	Intracellular concentration of Q_L_ mRNA
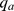	Intracellular concentration of Anti-Q RNA
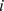	Intracellular concentration of iCF10
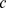	Intracellular concentration of cCF10
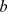	Concentration of PrgB membrane protein
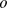	Ration of plasmid DNA in repressed form
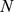	Plasmid copy number, equal to 5
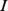	Extracellular concentration of iCF10
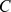	Extracellular concentration of cCF10
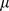	Specific growth rate, equal to 0.0002567 (1/s)
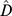	Number of donors
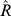	Number of recipients
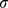	Volume conversion factor
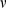	Cell volume
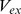	Extracellular volume

**Table 3:** Parameter values of pCF10 system.

Reaction constant	Name	Value	Unit
***K*** **_1,1_**	transcription rate of *prgQ*, DNA in repressed conformation	0.0084	(nM/s)
***K*** **_1,2_**	transcription rate of *prgQ*, DNA in active conformation	0.0876	(nM/s)
***K*** **_1,3_**	transcription rate of Anti-Q, DNA in repressed conformation	0.0125	(nM/s)
***K*** **_1,4_**	transcription rate of Anti-Q, DNA in active conformation	0.0014	(nM/s)
***K*** **_1,5_**	generation rate of PrgB	0.01	(1/s)
***K*** **_1,6_**	generation rate of extracellular iCF10	0.005	(1/s)
***K*** **_1,8_**	generation rate of extracellular cCF10	0.12	(nM/s)
***K*** **_2,6_**	importation rate of iCF10	0.001	(1/s)
***K*** **_2,8_**	importation rate of cCF10	2.57×10^−4^	(1/s)
***K*** **_3,5_**	equilibrium constant of Q_PRE_ and Anti-Q reaction	0.0443	(1/nM)
***K*** **_3,8_**	equilibrium constant of DNA binding reaction	1.00×10^6^	-
***K*** **_4,1_**	degradation rate of Q_s_ mRNA	0.001	(1/s)
***K*** **_4,2_**	degradation rate of Q_L_ mRNA	0.001	(1/s)
***K*** **_4,3_**	degradation rate of Anti-Q RNA	1.36×10^−4^	(1/s)
***K*** **_4,6_**	degradation rate of intracellular iCF10	1.00×10^−6^	(1/s)
***K*** **_4,8_**	degradation rate of intracellular cCF10	1.00×10^−6^	(1/s)
***K*** **_4,9_**	degradation rate of PrgB protein	1.00×10^−6^	(1/s)

The exponent on 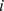 in the expression for 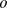 is taken to be four because four peptides bind to the protein tetramer to manipulate the configuration of plasmid DNA [44]. The first three rows of the column vector 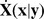 represent the net rates of formation of Q_s_ RNA, Q_L_ RNA, and Anti-Q RNA. The total transcription rate of Q_PRE_, 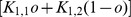, includes the rate 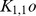 for plasmid DNA in repressed form and the rate 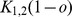 for the active form; the total generating rate should be proportional to plasmid copy number 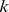. If Q_PRE_ is bound with Anti-Q, it becomes Q_s_, otherwise it becomes Q_L_; the fraction of Q_PRE_ to Q_s_ is given by 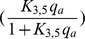 and to Q_L_ by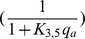. The rate constants for the degradation of Q_s_ and Q_L_ are 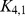 and 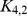, respectively; the terms containing the growth rate 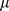 represent dilution of intracellular entities due to growth. Similar to the generating rate of Q_PRE_, 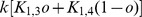 describes the generation rate of Anti-Q. The second term of the third row represents the consumption rate of Anti-Q due to binding with Q_PRE._ The uptake rate of iCF10 or cCF10 is proportional to its extracellular concentration. The final row is the mass balance of PrgB with its production rate assumed to be proportional to the concentration 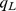 of Q_L_.

The generation and uptake rates of extracellular iCF10 or cCF10 are described in 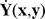 of Eq.(2). The first term of the first row of 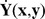 describes the rate at which extracellular iCF10 is translated by Q_s_ RNA or Q_L_ RNA in donor cells, and the first term of the second row indicates the rate at which extracellular cCF10 is produced by recipient cells. The second term of 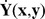 represents the uptake rate of iCF10 in row 1, and the uptake rate of cCF10 in row 2.

The derivation of extracellular concentration equations (Eq.(2)) are from the extracellular equations formulated in particle number shown in Eq.(3) where 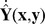 is the change of extracellular particle number. In terms of the notation for particle numbers, the change in the extracellular environment is written as
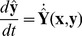
(3)



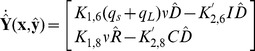
, 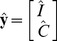


There are two kinds of reactions in 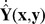, formation and transport. The formation is described by particle number generated per cell per unit time multiplied by cell number. The transport rate is proportional to the product of the extracellular concentration and cell number. Note that 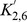 or 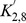 is not a constant because the uptake of iCF10 occurs by active transport at a rate depending on PrgZ protein [37]. By assuming the particle number of PrgZ to be proportional to cell volume 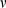, 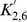 or 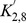 can be represented as 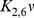 or 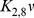 where 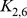 and 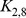
are constants. The formation of cCF10 is proportional to the number of recipient cells. Dividing Eq.(3) by V_ex_which is assumed to be a constant, we have equations for extracellular concentrations, Eq.(2). The cell numbers for donors and recipients for planktonic circumstance are described by Eqs. (4) and (5) below where 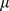 is the specific growth rate.
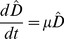
(4)

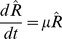
(5)


Note that we don’t account for conjugation in this study so that the change of cell number only comes from exponential growth.

#### PBM with Stochastic Intracellular Gene Regulation, for Planktonic Environment

The system of interest can be better described by the population balance equation (PBE) coupled with the extracellular environmental equations. A generic formulation of PBE is presented by Ramkrishna [50]. It distinguishes a vector of internal coordinates 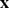 and a vector of position coordinates 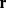; the former represents quantities associated with the cell and the latter denotes the location. Cells with the same coordinates are viewed as indistinguishable. Note that the position coordinate is not needed for well-mixed planktonic environment but is necessary for biofilm modeling. The formulation of a PBE with intracellular stochastic processes described by continuous variables in Ito stochastic differential equations is introduced in our previous work [51]. In this study we formulate a PBE with discrete intracellular states.
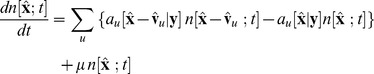
(6)


The PBE for planktonic circumstance is shown in Eq.(6) where 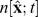 is the number of cells with state 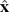
(symbols with caret tops represent particle numbers). The 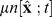 describes the rate of increase of cell number due to replication. The particle numbers of intracellular species are related to concentrations by 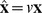 where 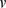 is the cell volume and 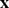 represents concentrations assumed to be uniform within the cell, 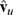 the vector describing the stoichiometric change of 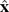 and 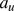 the propensity [52], [53] associated with reaction 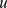.

It is convenient to also have PBE with intracellular states in concentration 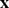
_._ Thus we set 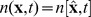. We further use the notation 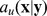, the propensity represented in terms of concentrations of the 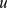 ^th^ reaction, as it is different from 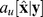. The relationship between 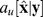 and 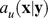
is elucidated well in the literature [26]; the effect of dilution on intracellular variables of concentration is lumped into the degradation rate. Thus the version of Eq.(6) written in terms of concentration is given by
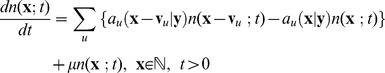
(7)where the equation implies the daughter cells share the same intracellular concentration as parent cells.

The extracellular equation is identified as Eq.(8)
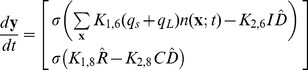
(8)


The extracellular variable 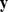 may be viewed as deterministic as the stochastic exchange rate with the numerous cells in the well-mixed environment can be regarded as averaged. Also, we know 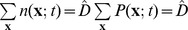 where 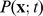 is the probability of a cell with state 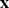 at time 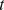.

While doing simulation of Eq.(7), the DNA conformation change together with the sense-antisense interaction between Q_PRE_ and anti-Q are considered as fast reactions. A quasi-steady-state assumption is applied to the chemical master equation for calculation purposes by separating variables 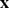 into 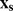 and 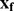, the slow and the fast reaction species. The probability can be described by 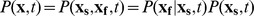 with 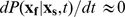, and the master equation for calculation solely in terms of 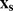. The propensity of 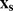 can be approximated by 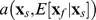 [54]. The reactions and propensities for stochastic simulation are listed in Table 4.

**Table 4:** The reactions and propensities for stochastic simulation.

Reaction	Description	Propensity
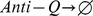 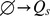 **or**	Generation of every Q_s_ occurs with consumption of one Anti-Q	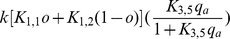
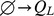	The generation of Q_L_	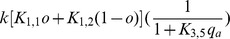
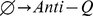	The generation of Anti-Q	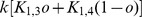
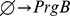	The generation of PrgB	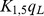
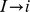	The uptake of iCF10	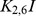
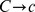	The uptake of cCF10	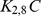
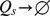	The degradation of Q_s_	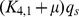
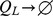	The degradation of Q_L_	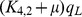
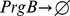	The degradation of PrgB	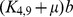
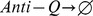	The degradation of Anti-Q	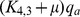
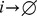	The degradation of iCF10	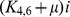
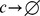	The degradation of cCF10	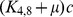

### Modeling of Biofilm Environment

**Figure 2:**
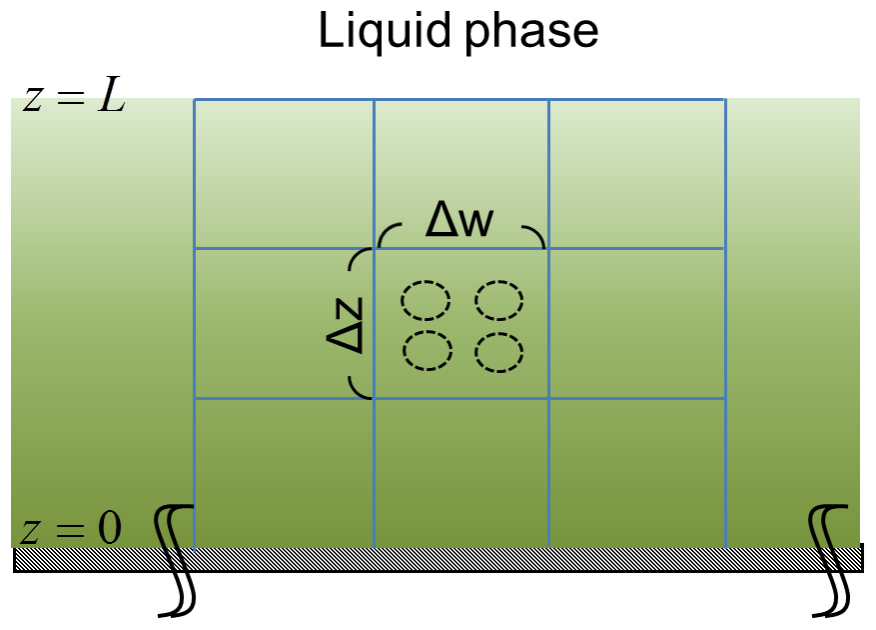
The schematic representation of the biofilm model. The width of biofilm is considered to be much larger than its thickness (L). For vertical direction, one end of biofilm attaches to wall (z = 0) and the other end is exposed to liquid phase (z = L). The biofilm is discretized into many subdomains, denoted by the boxed regions. The length of subdomain is considered to be small enough for applying well-stirred assumption.

In modeling the biofilm environment, we envisage a two dimensional film with vertical and horizontal coordinates (see Figure 2). The top of the film is exposed to a well-mixed fluid environment with concentrations of signaling molecules maintained constant while the bottom of the film is impervious to their transport implying a zero gradient boundary condition. We will identify the population balance equation for the cells and the mass balance equations for the environment at each point in the biofilm. The cells are assumed to be sessile and uniformly distributed throughout the film. The mechanism of gene regulation and the kinetic constants are assumed to be the same as for the planktonic environment. We assume no movement or translocation of cells in biofilm as the bacteria are trapped within the extracellular matrix. We analyze a well-developed biofilm with constant thickness and porosity [55]. The detachment of cells from biofilm is assumed to be a continuous process having no effect on extracellular structure or arrangement of cells.

For computational purposes, it is convenient to divide the entire domain of the biofilm into a two-dimensional grid of subdomains. The equations written for any points in the film are then adapted to the subdomains (for both deterministic and stochastic models). Each subdomain is suitably small (the length of the compartment is less than 5 µm) to consider the extracellular variables to be well mixed. The molecular dimension of iCF10 or cCF10 is 29×15×14 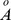 [49]. The diffusion coefficient 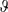 is approximated by the Stokes-Einstein equation with correction for the biofilm environment [56] affording a value of 110.28 µm^2^/s. Comparing to the reactions, the rate of diffusing through the compartment is 10^2^–10^6^ times faster.

#### The deterministic model for biofilm circumstance

We consider a biofilm of two dimensional domain 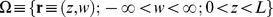 which represents a rectangular extent with position vector 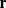 comprising a vertical coordinate 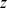 and a horizontal coordinate 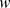. The biofilm is exposed to a well-stirred fluid at 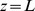, while the bottom 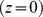 is impervious to transport of any chemical species. The population of cells is described by a number density 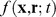 in spatial and internal coordinates representing concentration of intracellular variables. The population balance equation for this situation is given by
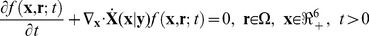
(9)


The above equation is coupled to environmental equation which accounts for diffusion of extracellular species and their exchange between the environment and the cells.
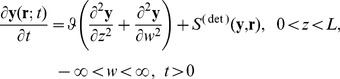
(10)where



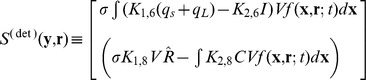



is the local rate of change of extracellular variables due to cells and 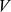 is the volume of 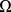 (with unit length in third dimension). Eqs. (9) and (10) must be supplemented with boundary and initial conditions. For the population density we have the initial condition
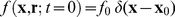
(11)which implies that all cells have the same initial state and that the number density is 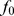 everywhere and 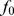 can be determined by



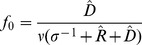
(12)The initial and boundary conditions for 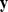 are given by
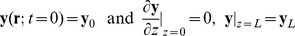
(13)where 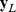 is the concentration of 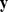 in fluid phase above the biofilm. The vectors 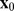 and 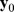 are specified in Eqs(1) and (2). Boundary conditions with respect to 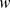 are omitted in favor of periodic boundary conditions in a discretized domain of the biofilm. The thickness, 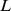 of the biofilm is assumed to be 240 µm [57]. For computational purposes, the biofilm domain 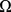 is discretized into a two-dimensional grid of subdomains identified by a single integral index 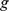. Thus we let



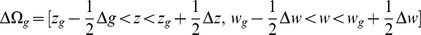
where 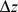 and 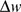 represent the vertical and horizontal lengths respectively of each subdomain. The subdomain 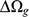 has volume 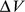 with its centroid at 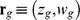. The subdomains are all of equal volume so that 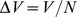
_,_ where 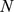 is the number of subdomains. We adapt Eq.(9) to the subdomain 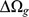 with due apologies for use of the same symbol 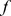 for the number density in the spatially integrated form



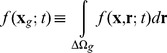
which is the number of cells per unit volume of intracellular state space. On the left hand side above, we have renamed the intracellular vector 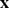 as 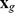 to indicate that the cells in subdomain 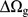
should be distinguished from cells from other subdomains. Integrating Eq.(9) over 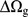 we obtain
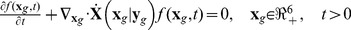
(14)where 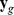 is the averaged extracellular concentration within the subdomain with components 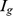 and 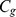 (the uptake rate of peptides is linear to extracellular concentration).

The extracellular concentration within each subdomain is ready to be solved by 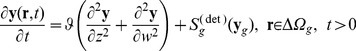
(15)


with
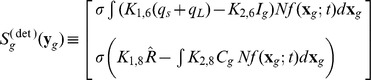
which is the rate of change of extracellular variables in the subdomain 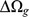
due to cells. Eq.(15) is calculated by finite difference of each subdomain with continuity concentration and flux on the boundary of subdomain. The simulation is carried out for every subdomain at each time step.

#### The stochastic model for biofilm circumstance

We formulate the stochastic model by using Reaction-Diffusion Master Equation (RDME) [58] which accounts for both intracellular stochastic processes and extracellular stochastic diffusion. Note that the well-mixed assumption allowing us to separate the extracellular equation from intracellular stochastic processes is no longer valid in the biofilm case. The basic concept of RDME is to grid the system volume 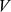 into the sub-volumes and treat the diffusion of particles from one compartment to another as random walk which can be considered as a first order reaction in the master equation [58]. We first partition the system into compartments each comprising exactly one cell. For a system with 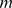 donor cells, we define 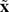 as the composite vector of intracellular variables with 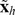 the intracellular states of a cell in compartment 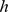. Similarly, 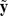 represents the composite vector of extracellular variables with 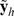 denoting extracellular variables in compartment 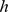, and 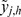 is the 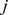 ^th^ element in 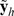. Thus we have
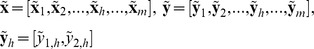
where 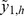 is iCF10 and 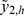 is cCF10. Next, we formulate RDME, Eq.(16), which allows us to trace all intracellular and extracellular variables in every compartment.



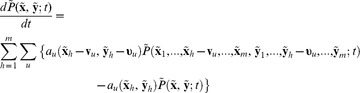


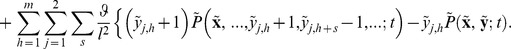


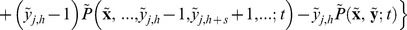
(16)where 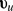 is the vector describing the stoichiometric change of 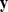 associated with reaction 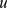, the sum over 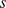 representing the net diffusional exchange with the immediate neighborhood compartments, and 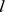 is the length of the compartment.

As shown above, the diffusion is much faster than reaction, thus we may apply quasi-steady-state approximation to the extracellular variables in the stochastic model. In other words, the distribution of 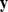
may be assumed to be stationary immediately with respect to dynamic changes in 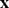 [54]_._ Thus.
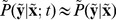
(17)


We rewrite Eq.(16) in terms of the conditional probability as shown in Eq.(18) below 
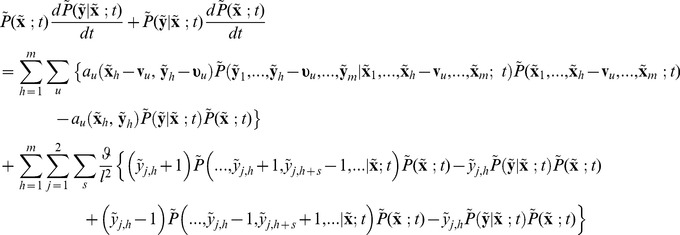
(18)


We apply the approximation in Eq.(17) to Eq.(18) and sum over all 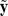
to obtain.
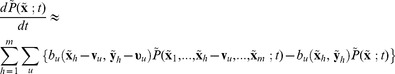
(19)where 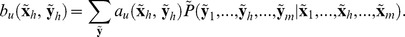


The uptake rate of extracellular species is first order with respect to concentration so that the reaction propensity 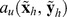
is linear with respect to 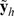 and, in view of rapid diffusional homogenization within the compartment, we have 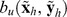 as 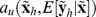 [54]. Then, we rewrite Eq.(19) as.
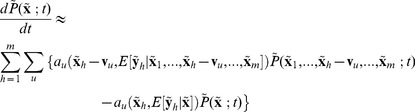
(20)


Note that 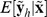 is stripped of its temporal dependence because of Eq.(17).

Eq.(20) couples together the probability of all states in every cell and is very expensive for computation due to a large number of states. We have therefore further simplified Eq.(20) by summing over all 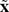 except 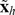 to yield an equation in the probability distribution at time 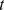 for intracellular states in only compartment 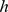, which we denote by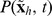.
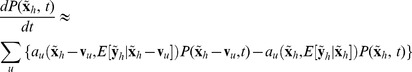
(21)


In Eq.(21) the expectation of 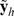 is conditional only on specification of 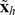 as account has been taken of the dependence on all other 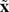
’s.

For computation, we enlarge the compartment so that we have a total of 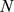
compartments in the system. Similar to the deterministic model, we adapt Eq.(21) in the number of cells to the subdomain 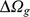 by defining 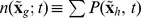 which sums over all 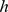 compartments within subdomain 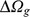.
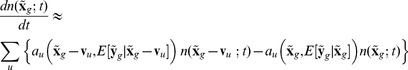
(22)


The system is simulated by 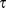-leap [59] method. However, instead of choosing a minimum value of 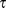 at each step, we use a fixed 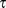 for every step. Although it forces us to choose the smallest 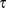, we take advantage of not having to calculate 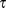 at each round. Note that the simulation calculates a sample path instead of a distribution. The mean value at 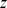 location shown in result section is obtained by averaging along with 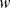 coordinate.

Similar to 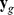 of the deterministic model, 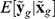 in subdomain 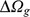
can be obtained by averaging 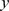 calculated by equation below.
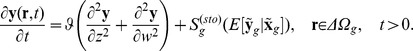
where



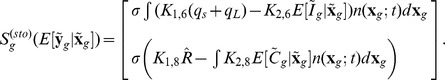



describes the change of extracellular variables due to cells. The diffusion equations of both stochastic and deterministic model use Alternating Direction Implicit (ADI) finite difference method [60]. But, the exchange between cells and the environment is calculated explicitly because there is no implicit method for tau leap model.

#### Comparing deterministic and stochastic models for biofilm circumstance

For biofilm (figure 2), the deterministic model predicts the same value for different 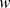
(horizontal coordinate) as long as 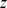
(vertical coordinate) is fixed. For the stochastic model, due to randomness, cells in different 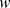 may have different intracellular states. Thus, we average the result from the stochastic model along with 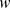 and compare the prediction of the deterministic model at the same 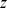 position.

## Results

### Biofilm Changes the Nature of Intracellular Stochasticity

Instead of directly measuring the successful events of plasmid transfer, many experiments monitor the expression of *prgB* [37]–[41], [61]. In this study, the PrgB protein concentration is one of the intracellular states and serves as an indicator of conjugation.

**Figure 3:**
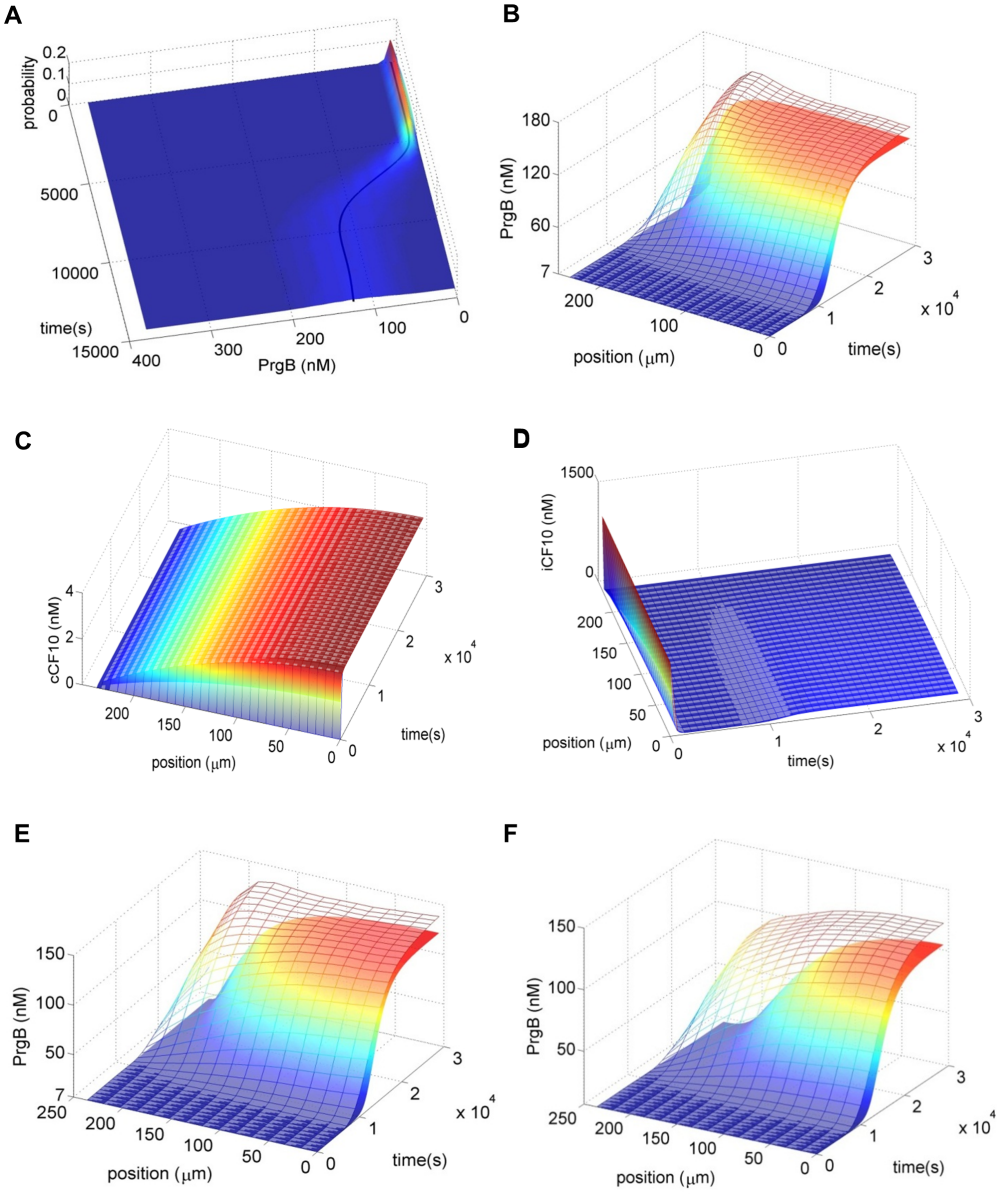
The behaviors of cells. A) For planktonic environment, the probability distribution of PrgB protein from population balance model with intracellular stochastic gene regulation (surface) and the averaged value estimated by the deterministic model (solid curve) are consistent. B) For biofilm cells, the bulk concentrations of both iCF10 and cCF10 are set to zero as in the natural niche of *E. faecalis*, the bulk fluid continues flowing [68]. The probability distribution of PrgB protein with time from deterministic model (solid surface) deviates from that of stochastic model (mesh surface) because the former is not able to describe the difference of stochasticity from planktonic to biofilm environment. C) Both stochastic and deterministic models have the same extracellular cCF10 concentration. D) Although the extracellular iCF10 predicted from the deterministic model is slightly lower than that of stochastic model around time equal to one hour, this should not be the reason causing deterministic model predicting lower value of PrgB in figure 3B because lower iCF10 should lead to higher PrgB. E) and F) Increasing particle number of iCF10 results in more deviation of the deterministic model. The bulk concentration of iCF10 is 10 nM for figure 3E and 30 nM for figure 3F. The bulk concentration of cCF10 is 0 nM for both. When bulk concentration of iCF10 increases, the particle number of intracellular iCF10 also increases and the fluctuation of intracellular iCF10 is supposed to be less. But a higher deviation of PrgB protein from the deterministic model is observed.

In planktonic environment (figure 3A), the prediction of the deterministic model (Eqs. (1) and (2)) is consistent with the average from the stochastic model (Eqs. (7) and (8)). This result is not surprising since the deterministic model has been used for many decades and does predict the average behavior in numerous situations. However, it treats all intracellular states as continuous variables and ignores the natural discrete character of particle copy number. Therefore, its universal applicability is at stake, especially in biological systems with low intracellular particle number. Whenever particle number is too low to be treated as a continuous variable, the deterministic model could misrepresent the real system. On the other hand, the stochastic model based on CME does not suffer from this shortcoming as it directly deals with discrete numbers of reacting species.

In biofilm environment (figure 3B), interestingly, deviation is observed between the deterministic model and the stochastic model. This phenomenon is more pronounced near the surface; the bottom of the film is at 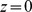
(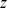 is vertical coordinate, figure 2) and the outer surface at 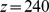. The average values of the stochastic model shown in figure 3 B are obtained by averaging through the horizontal coordinate at the same vertical position. In order to further ensure that the deviation does not arise from a different extracellular environment, we examine the extracellular concentrations of cCF10 and iCF10. For extracellular cCF10 (figure 3C), the two surfaces overlap so that the deviation does not arise from it. For extracellular iCF10 (figure 3D), although minute differences can be observed it is not the reason for lower PrgB protein of the deterministic model because less iCF10 should lead to higher PrgB protein.

### Increasing Particle Number does not Grant the use of Deterministic Models

For the biofilm circumstance, there are two major reasons identified in this study, which cause deviation of the deterministic model from the stochastic model. From literature [62], smaller particle numbers are known to lead to larger stochastic fluctuations so we first discuss the effect arising from particle number. Without washing out from mass transfer of flowing bulk fluid, cells maintain high extracellular concentration of iCF10 and cCF10. But, in biofilm, extracellular particles are exchanged with flowing fluid phase resulting in low intracellular particle number. To see the effect of particle number, we increase the bulk concentration of iCF10 but keep that of cCF10 the same (figure 3E and 3F). Interestingly we observe the deviation of the deterministic model becomes larger when the concentration of iCF10 is increased. This observation is not consistent with the prevailing impression that increasing particle number of a control variable leads the system to the deterministic limit. Instead, this result suggests that increasing particle number does not always grant the use of deterministic models.

### The Stochastic Effect of Gene Regulation is Complicated and Influenced by all Variables

In the other side, increase bulk concentration of cCF10 indeed reduces the deviation of the deterministic model (figure S1). It is our contention that the contrasting effects of cCF10 and iCF10 on the relationship between the deterministic model and the stochastic average are a manifestation of the same phenomenon to be elucidated below. The influence of cCF10 or iCF10 on gene regulation is through DNA conformation. Based on the fact that iCF10 makes DNA in repressed configuration but cCF10 changes it to the active configuration, we define below the following probabilities for the stochastic model.

Pr (pCF10 in repressed configuration)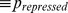 (23)

Pr (pCF10 in active configuration)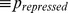 (24)

In the other side, the deterministic model follows 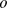 in Eq.(1). While 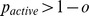, higher value of PrgB is predicted by the stochastic model (figure 3B–F and figure S1).

The above difference between deterministic and stochastic models comes from the fact that the average of a nonlinear function is not equal to the function of the average. The phenomenon has been recognized for decades [52]. The analytical approach by Van Kampen [63] provides the primary understanding. From system size expansion, the bigger the size of the system, the less pronounced is this phenomenon. Paulsson et al. [29] investigates with stochastic simulation algorithm (SSA) and conclude that this phenomenon is profound while particle number is low. In addition, a biological implication has been proposed and named as stochastic focusing. Stochastic focusing can be understood as follows. The signal noise itself may amplify the effect of the signal. Of course, it is true only if the particle number of signaling molecule is low enough. The original stochastic focusing proposed by Paulsson is for signal noise so that it *cannot* be applied to our system in which the extracellular fluctuation of the signaling molecule is averaged out as described in the section Models. In our study, only the effect of intracellular stochasticity has been investigated. Nevertheless, the concept of stochastic focusing as originally envisaged is the same as that implied in this work, viz., through stochastic fluctuation, there is an attempt by cells to “amplify” the effect from species with low particle number. Therefore, *we further extend the use of the term, stochastic focusing, to describe this underlying concept*.

By applying the foregoing concept, for low particle number of cCF10, the stochastic focusing of cCF10 may result in 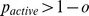. Conversely, for the system with low particle number of iCF10, the stochastic focusing of iCF10 results in 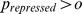. Indeed, for a single variable, the effect fades out by increasing particle number but the behavior of a cell is decided by the overall effect. When particle number of iCF10 and cCF10 are both low, both stochastic focusing is high and the outcome depends on which effect is bigger. We know that cCF10 facilitates conjugation while iCF10 suppresses it. If the stochastic focusing of cCF10 is larger, the PrgB predicted by the stochastic model is higher than that of the deterministic model and this is the case in figures 3; when increasing particle number of iCF10 (decrease stochastic focusing from iCF10) the stochastic focusing of cCF10 become more significant and the deviation of the deterministic model is larger (figure 3 E and 3F). It is also possible to let stochastic focusing of iCF10 dominate the system and the deviation of the deterministic model shows in opposite direction (figure S2); under this circumstance, the deviation of the deterministic model is seen to increase as bulk concentration of cCF10 is increased.

### Stochastic Focusing is Amplified by Interrupting the Feedback Loop

We have demonstrated that the change of particle number alters stochastic focusing, but the influence of extracellular mass transfer on stochastic focusing is not through it alone. We propose here a new idea that interrupting the natural feedback loop can also be a major cause for deviation of the deterministic model. To illustrate it, we compare steady-state values for two cases. For both cases, both iCF10 and cCF10, the periodic boundary condition is applied to horizontal coordinate (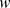) and the reflection boundary condition is applied to vertical coordinate at the bottom of biofilm (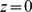). In the first case, we allow cells to control the extracellular iCF10 by assigning a reflection boundary condition at the top of biofilm; and in the second case, the bulk concentration of iCF10 is fixed. Because the purpose is to see the feedback effect of iCF10, reflection boundary is applied to cCF10 at the top of biofilm for both cases.

With reflection boundary condition at the top of biofilm, the cell concentration used above shows only a small difference between the stochastic average and the deterministic result so we simulate the case of 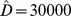, 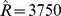, and 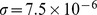. For the first case, the iCF10 concentration calculated by the deterministic model at the top of the film is 2493 nM and we assign this value as boundary condition for second case.

**Figure 4:**
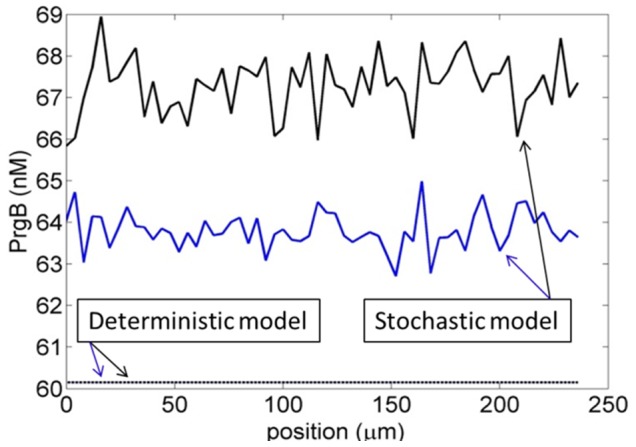
The influence of interrupting feedback loop. The dash represents the prediction from the deterministic model and the solid line from stochastic model. The black color is for the system with given bulk concentration as boundary condition and the blue color is for system with reflection boundary condition. For the deterministic model, the results of two cases are overlapped. For stochastic model, lack of feedback causes higher stochastic focusing.

The predictions of the deterministic model for these two cases overlap (figure 4). However, a clear difference can be observed for the predictions of the stochastic model. When cells cannot fully control extracellular iCF10 and feedback is intercepted, they have less ability to compensate for the influence of random uptake of cCF10. Note that the feedback loop attenuating stochastic fluctuation does not contribute notably because of the large number of cells averaging the extracellular iCF10 concentration. Moreover the iCF10 concentration is more than 10^3^ nM and its stochastic fluctuation should not be significant. The outcome mainly results from cells that are not allowed to balance the stochastic focusing from cCF10 by controlling the production of iCF10.

### Intracellular Stochasticity Renders Cell Population Less Sensitive to Environmental Fluctuation

**Figure 5:**
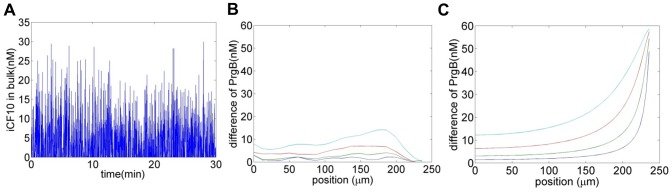
Cells with intracellular stochasticity are less sensitive to small environmental fluctuation. When iCF10 concentration in liquid phase is subject to small environmental fluctuation, stochastic model are less sensitive to it. A) The Gaussian white noise with standard deviation of 10 nM. Only positive concentration is taken from the noise (the negative concentrations have been set to zero) and exactly the same noise is imposed to both models. B) and C) the response from stochastic and deterministic models, respectively, after exposing to different magnitude of Gaussian white noise, blue 10 nM, green 20 nM, red 40 nM and cyan 80 nM. Cell population may probably use this feature against unwanted environmental fluctuation.

In the planktonic case, small environmental fluctuation may influence the system just a little as the concentration of extracellular iCF10 is of the order of 10^3 ^nM and that of cCF10 is of the order of 10^2^. However, in the biofilm case, the concentrations of extracellular species are nearly zero. It is of interest to understand how the cell population responds to environmental fluctuations. In this study, a Gaussian white noise is imposed to the bulk concentration of iCF10 (figure 5 A).

It may be possible that the cell population uses intracellular stochasticity to minimize the influence of environmental fluctuation. We use deterministic and stochastic models to analyze the behavior of cells. The PrgB level for the biofilm case with bulk concentration of iCF10 and cCF10 as zero is served as the base for examining the response of cells to small environmental fluctuation. The difference of PrgB shows how cell population responds to small environmental fluctuation (Figure 5B from the stochastic model and 5C from the deterministic model).

That environmental change has less effect on cells deeply inside the biofilm, is consistent with commonly held belief to this effect [64]. For cells close to the surface, we observe that the stochastic model is much less sensitive to environmental change. The difference between Figure 5B and 5C comes *only* from the intracellular stochasticity (as described in the section Models). In other words, the stochastic nature of intracellular gene regulation may help to reduce the influence of small environmental change on the cell population. The underlying reason is due to the fact that cells attempt to “amplify” the effect from signal while its concentration is low. To further explain this concept, we refer to the prediction from the deterministic model as 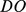 (deterministic output), that from the stochastic model as 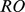 (real output) and define stochastic focusing 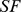 as the difference between 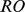 and 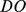. For a given 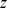 position, we may write.
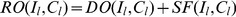
(25)where 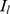 and 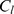 denote iCF10 and cCF10 concentrations in liquid phase (as indicated by suffice 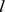) respectively. We have equation below, as an example, to describe the change of 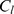 from zero to one.



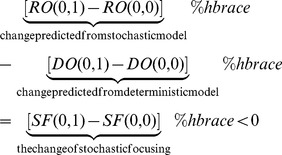
(26)Clearly, 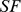
decreases as 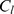 increases so the change of 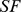
is in the opposite direction to that of 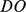. Due to this feature, the change predicted from the stochastic model with respect to increasing environmental cCF10 is less than that of the deterministic model. Similarly, when 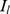 is increased, the stochastic model shows less sensitivity to the change (figure 5). Cell population may utilize intracellular stochasticity against small environmental change. Thus, the response of cells is controlled more by their own density.

## Discussion

In this study, we have investigated using a layer model the behavior of a biofilm whose environment is altered by mass transfer with a bulk liquid phase. We have emphasized the role of intracellular stochasticity and investigated the fundamental concept causing the deterministic model to deviate from observation. Most models for biofilm growth usually focus on the biofilm structure, and extracellular mass transfer [15], [65]. Few of them discuss the stochasticity of intracellular gene regulation. Thus the issue of stochastic focusing demonstrated here is overlooked in the literature.

Although the layer model does not fully reflect the structure of biofilm, nor include the movement of cells within the biofilm [6], it provides an appropriately simple setting for demonstration of the effect of extracellular mass transfer on intracellular stochasticity that cannot be handled by a deterministic model. Therefore, the deterministic model possibly deviates from the stochastic model as the system is subject to extracellular mass transfer. The concept proposed by this study is ready for application to other mathematical biofilm models because all of them involve mass transfer. Of course, the structure of biofilm or movement of cells can affect the stochastic focusing. But, as long as the particle number is low and the feedback loop is interrupted, the stochastic focusing should still be pronounced. With incorporation of the additional features of biofilm structure and of cell movement, and considerably augmented computational power, the formulation and methodology of this paper would help to discover stochastic focusing in this more complex setting. The simplifying assumptions of this paper, made it possible however to discover the basic attributes of stochastic focusing.

We have identified two main causes by which mass transfer alters the stochastic nature; (i) by interrupting the feedback loop and (ii) reducing the particle number. For (i), this study illustrates the concept that feedback loops playing an important role on stochastic focusing. The example demonstrated in the results section may not closely purport to a specific biological system but nevertheless the predictions shed a light on the above concept. For example, there are two different experimental protocols of biofilm formation in *E. faecalis.* Protocol 1 is to inoculate cells in a 96-well microtiter plate [66]. Protocol 2 is to place a plastic “coupon”, which provides a flat surface for cells to attach, in a stirred bioreactor. The “coupon” appears like a coin about 1 cm in diameter; the chemostat is more than a liter with continuous feeding and removing medium [61]. Case 2, discussed in the results section may serve well to describe the circumstance of protocol 2. As for protocol 1, the concentration of iCF10 in 100 uL medium may be influenced, to some degree, by the cells. Protocol 1 is closer to case 1 (in the results section) than case 2. Therefore we may surmise that protocol 1 may have less stochastic focusing as compared to protocol 2. For (ii), we have pointed out that the stochastic effect of gene regulation is an overall outcome of the reaction network, exchange with the environment, and transport. Hence it is important to recognize that behavior obtained by indiscriminately increasing particle number of a control variable does not necessarily submit to deterministic modeling as it may result in even larger deviation. The simplicity of the deterministic model must be weighed with losing the detail of the nature of stochasticity. In this connection, various situations in which stochasticity may be important and cannot be addressed by the deterministic model, have been discovered recently (from literature [27]–[34] and from this study). These studies provide us a better sense of direction towards weighing computational cost with modeling detail.

We have recently reported the conjugation of pCF10 as a quorum sensing system with dual signaling molecules for self sensing and mating sensing [67]. This dual signal system allows cells not only to sense the density of recipients but also donors. Undoubtedly, sensing both the population of donors *and* recipients is critical to survival. However, for *E faecalis*, biofilm in situ may grow in the presence of flowing bulk fluid [68] and the concentration of signaling molecules is sensitive to environmental fluctuation. Without appropriate mechanisms, the decision of conjugation may depend majorly on the noise instead of cell density, especially for cells near surface. The model development in this study suggests that the stochastic nature of intracellular gene regulation may render the cell population less sensitive to environmental fluctuation (figure 5). Cell population may use regulation to minimize the influence from extracellular noise so that cells can sense their own population and ignore the surrounding fluctuation. From the aspect of evolution, it has been experimentally shown that cells are able to adjust the intracellular stochasticity for survival [32]. It is therefore possible that this delicate mechanism of utilizing intracellular stochasticity is the product of evolution.

In pCF10 biofilm system, extracellular iCF10 and cCF10 are manipulated by externally controlling their concentrations in the fluid phase so that we can clearly illustrate experimentally a picture of the concept. We have not discussed the stochastic focusing from other intracellular variables in the result section to avoid confusion. When the stochastic focusing of iCF10 and cCF10 are small, the influence from other intracellular variables may be observable. Stochastic focusing is a complicated phenomenon with many reactions contributing to it. Although we show in this study that stochastic focusing in planktonic growth is negligible, it does not imply that stochastic focusing is not important in other planktonic systems. It is possible to observe stochastic focusing in planktonic systems [29] but its effect in biofilm circumstance is usually more pronounced.

Last but not least, reaction Diffusion Master Equation (RMDE) is formidable for its extremely high computational burden; we have proposed a way to separate extracellular mass transfer from intracellular stochastic processes to formulate a PBE. The computational burden of PBE is much less than that of RDME so that PBE allows us to analyze the behavior of cells for a much longer time.

## Supporting Information

Figure S1 Increasing particle number of cCF10 results in less deviation of deterministic model. The bulk concentration of cCF10 is 1 nM, 2 nM and 3 nM for A, B, and C; the bulk concentration of iCF10 is 100 nM for all three figures. When bulk concentration of cCF10 is increased, the fluctuation of intracellular cCF10 is reduced and less deviation of deterministic model is observed (solid surface as deterministic model and mesh surface as stochastic model).
(TIF)

Figure S2 The overall stochastic focusing of iCF10 and cCF10. The bulk concentration of cCF10 is 3 nM for A and 5 nM for B; the bulk concentration of iCF10 is 80 nM for both. A) the stochastic focusing of iCF10 dominates the system for 180< z <240 µm (for 0< z <180 µm, the stochastic focusing of cCF10 dominate the system) B) When particle number of cCF10 is increased, the deviation of deterministic model for 180< z <240 µm becomes larger and the deviation of deterministic model for 0< z <180 µm changes sign because the stochastic focusing of cCF10 no longer dominates the system.
(TIF)
