# Supplementary figures and images for: Role of Intracellular Stochasticity in Biofilm Growth. Insights from Population Balance Modeling
Source: PLoS One. 2013 Nov 13;8(11):e79196. doi: 10.1371/journal.pone.0079196 (PMC3827321; doi:10.1371/journal.pone.0079196)

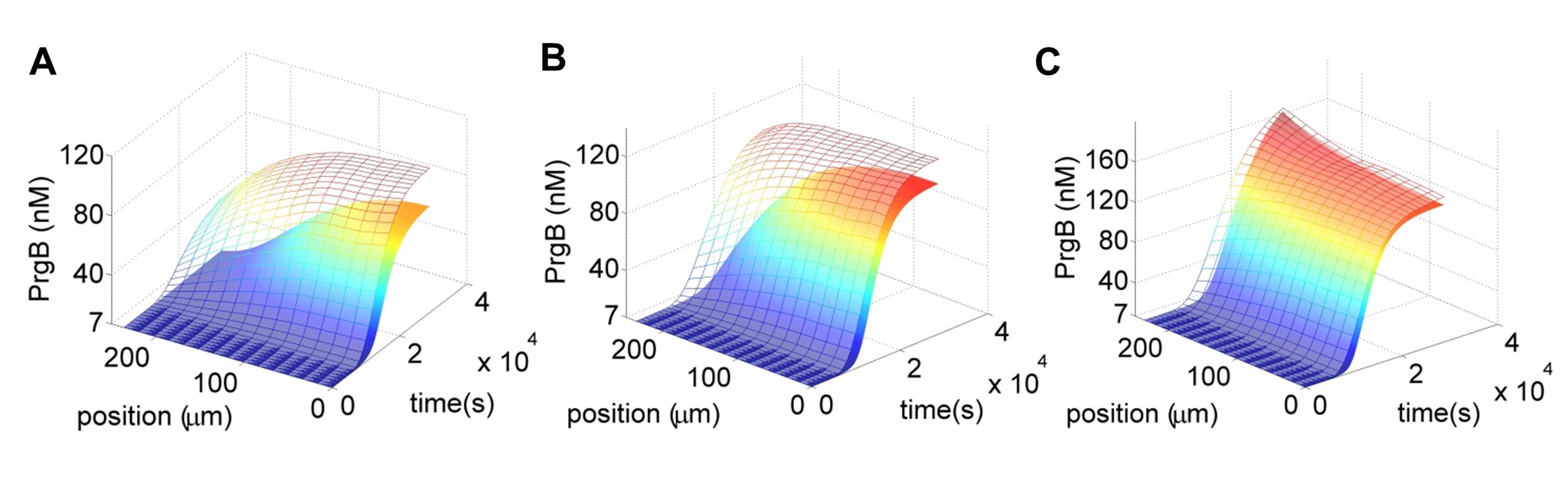

Supplement: Figure S1 — Increasing particle number of cCF10 results in less deviation of deterministic model. The bulk concentration of cCF10 is 1 nM, 2 nM and 3 nM for A, B, and C; the bulk concentration of iCF10 is 100 nM for all three figures. When bulk concentration of cCF10 is increased, the fluctuation of intracellular cCF10 is reduced and less deviation of deterministic model is observed (solid surface as deterministic model and mesh surface as stochastic model). (TIF) [file pone.0079196.s001.tif]

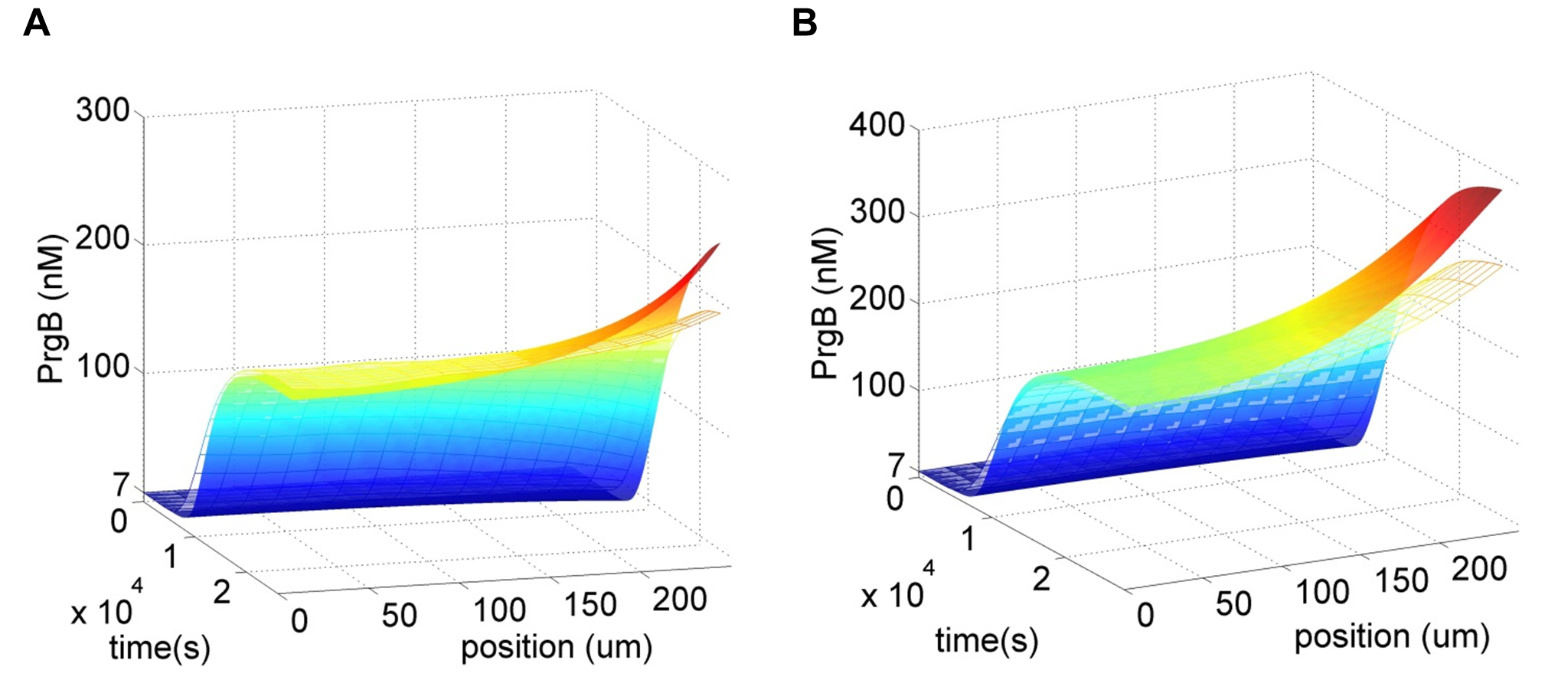

Supplement: Figure S2 — The overall stochastic focusing of iCF10 and cCF10. The bulk concentration of cCF10 is 3 nM for A and 5 nM for B; the bulk concentration of iCF10 is 80 nM for both. A) the stochastic focusing of iCF10 dominates the system for 180< z <240 µm (for 0< z <180 µm, the stochastic focusing of cCF10 dominate the system) B) When particle number of cCF10 is increased, the deviation of deterministic model for 180< z <240 µm becomes larger and the deviation of deterministic model for 0< z <180 µm changes sign because the stochastic focusing of cCF10 no longer dominates the system. (TIF) [file pone.0079196.s002.tif]
